# The Impact of Cybersecurity Practices on Cyberattack Damage: The Perspective of Small Enterprises in Saudi Arabia

**DOI:** 10.3390/s21206901

**Published:** 2021-10-18

**Authors:** Fawaz Alharbi, Majid Alsulami, Abdullatif AL-Solami, Yazeed Al-Otaibi, Meshaal Al-Osimi, Fahad Al-Qanor, Khalid Al-Otaibi

**Affiliations:** 1 Computer Science Department, Huraymila College of Science and Humanities, Shaqra University, Shaqra 11961, Saudi Arabia; 2 Computer Science Department, Community College-Shaqra, Shaqra University, Shaqra 11961, Saudi Arabia; malsulami@su.edu.sa; 3 Computer Science Department, College of Computing and Information Technology, Shaqra University, Shaqra 11961, Saudi Arabia; Alsolamiabdulatif@gmail.com (A.A.-S.); Yazeedsaad223@gmail.com (Y.A.-O.); Mesh3al144@gmail.com (M.A.-O.); jxjd757@gmail.com (F.A.-Q.); 5brh.net@gmail.com (K.A.-O.)

**Keywords:** cybersecurity, small enterprises, Saudi Arabia, data loss

## Abstract

Small and medium-sized enterprises represent the majority of enterprises globally and yet have some difficulties in understanding the impact that cybersecurity threats could have on their businesses and the damage they could do to their assets. This study aims to measure the effectiveness of security practices at small-sized enterprises in Saudi Arabia in the event of a cybersecurity attack. Our paper is among the first research papers to measure the effectiveness of cybersecurity practices and the threat posed by cybersecurity breaches among small enterprises in the event of cybersecurity attacks. A total of 282 respondents participated, all of them representing small-sized enterprises in Saudi Arabia. The study applies multiple regression tests to analyze the effectiveness of 12 cybersecurity practices in three aspects: financial damage, loss of sensitive data, and restoration time, at small enterprises. The findings indicate that having an inspection team and a recovery plan may limit the financial damage caused by cybersecurity attacks on small enterprises. The results also show that cybersecurity awareness, knowledge of cybersecurity damage, and professionals’ salaries were related to the loss of sensitive data. Furthermore, the results indicate that contact with cybersecurity authorities and having an inspection team have statistically significant effects on restoration time.

## 1. Introduction

The advancement of information and communication technologies has impacted many areas of our society, from online shopping to social interaction. Despite the positive impacts that such technologies have had, they also present many risks; for example, the use of information and communication technologies introduces the risk of cybersecurity attacks. A recent report estimated that the economic cost of cybersecurity attacks would reach more than $1 trillion worldwide for the period from 2017 to 2021 [1]. The same report indicated that two thirds of the organizations investigated had experienced some kind of cybersecurity threat in 2019. The cost of cybersecurity incidents goes beyond the direct cost to include various kinds of indirect harm, such as damage to the enterprise’s reputation, data breaches, etc. [2]. Thus, many organizations of different sizes have utilized technical and non-technical solutions to deal with cybersecurity threats.

Small and medium-sized enterprises (SMEs) play important roles in the economies of many countries. The majority of businesses are considered to be SMEs and they are responsible for a significant share of job creation. The World Bank estimated that seven out of ten jobs will be created by SMEs by 2030 [3]. SMEs are also responsible for more than 50% of employment worldwide [3]. Additionally, SMEs contribute more than 40% of the gross domestic product (GDP) in emerging economies [3]. Considering the importance of SMEs, the increase in the number of cybersecurity attacks targeting SMEs is alarming. For example, the percentage of cybersecurity attacks that targeted SMEs increased from 34% to 43% in the United States of America (USA) in 2015 [4] and increased again from 61% in 2017 to 67% in 2018 [5]. Consequently, to support SMEs in improving their cybersecurity practices and competencies, many authorities around the world have developed strategies and initiatives specially tailored to small businesses. The United Kingdom’s government developed a security guide to help small-sized organizations improve their cybersecurity practices [6]. Other countries also developed their own frameworks to promote increased awareness of cybersecurity threats such as by publishing the Framework for Improving Critical Infrastructure Cybersecurity in the USA [7] and instituting the National Agency for the Security of Information Systems (ANSSI) certification in France [8].

Measuring the impact of cybersecurity attacks on small businesses is an important matter that still requires further investigation. Although many research efforts have addressed cybersecurity education and awareness in small businesses [9], only a few studies have discussed in detail the impact of cybersecurity practices on the level of harm done to small enterprises by cybersecurity attacks [10,11]. Our research paper is among the first to analyze the impact of cybersecurity practices on the amount of damage resulting from cybersecurity attacks on small enterprises in Saudi Arabia. The aim of this research is to measure how certain security practices can affect small enterprises and the level of harm that may result from cybersecurity attacks.

This research tries to answer the following research questions:

- What is the impact of cybersecurity practices at small enterprises in Saudi Arabia in the event of cybersecurity attacks?
- What is the relationship between cybersecurity practices and the level of harm that may result from cybersecurity attacks?

The research questions can be reached through the following research objectives:

- To identify the current cybersecurity practices that can be used by small enterprises.
- To identify the possible harms that may result from cybersecurity attacks.
- To formulate a theoretical framework for determining the impact of various security practices on the harm caused by cybersecurity attacks, especially for small enterprises in Saudi Arabia.
- To conduct a survey to identify the relationship between cybersecurity practices and the level of harm that may result from cybersecurity attacks.
- To analyze the results of the survey using multiple regression analysis.

## 2. Background

The world is facing a high level of risk as new emerging technologies advance and improve. Cyber-attacks are considered a threat to individuals, businesses, and governments. They manipulate users to gain access to their information [12]. Many of the issues under the umbrella of cybersecurity relate to system applications, operating and communication systems, and electromagnetic equipment [13]. Cyberattacks can be defined as:

“A hostile act using computer or related networks or systems, and intended to disrupt and/or destroy an adversary’s critical cyber systems, assets, or functions. The intended effects of cyberattack are not necessarily limited to the targeted computer systems or data themselves—for instance, attacks on computer systems which are intended to degrade or destroy infrastructure or C2 capability. A cyberattack may use intermediate delivery vehicles including peripheral devices, electronic transmitters, embedded code, or human operators. The activation or effect of a cyberattack may be widely separated temporally and geographically from the delivery” [14].

**Figure 1:**
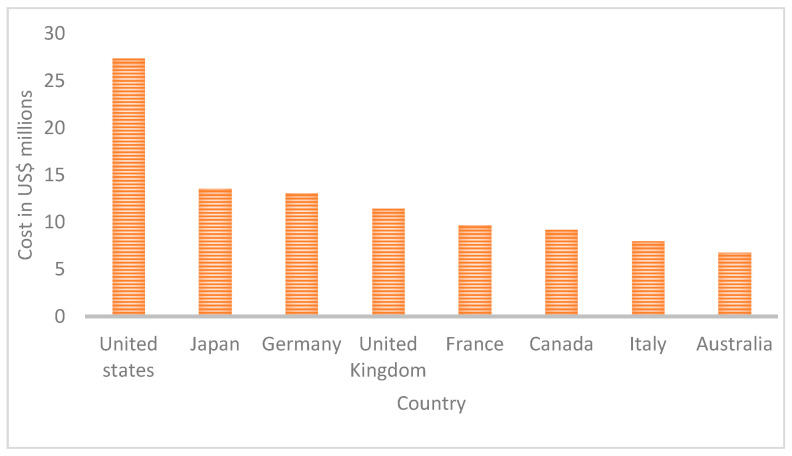
Total cost of Cyberattacks in various countries source [18].

In 2020, a report reviewed the main cybersecurity incidents that occurred in 2019 [15]. It indicated that more than 21 million unique passwords, and more than 770 million emails, had been hacked. It also pointed out that the details of 620 million web accounts had been stolen and offered for sale. In addition, more than half a billion Facebook accounts were unprotected from attack. Figure 1 shows the total cost of cyberattacks in various countries in 2018.

Saudi Arabia (SA) is one of the countries that has suffered the most from cyberattacks. The percentage of Saudi companies affected by cyberattacks increased from 19% in 2012 to reach 31% by 2018. Such attacks cost 2.6 billion SAR for the same period. One of the worst such crises to occur in the Kingdom of Saudi Arabia (KSA) was the 2012 attack on the Saudi Aramco oil company, which destroyed 30,000 computers [16]. Therefore, the cybersecurity market in the KSA is expected to increase from $2.9 billion in 2019 to $5.7 billion by 2023 [17].

A study conducted by [18] critically reviewed the definition of SMEs. It indicated that there is no universal definition for SMEs and that different terminologies are used for enterprises that are not considered large, including small businesses, small and medium-sized enterprises, and micro, small, or medium enterprises. Nevertheless, these terminologies are used interchangeably. The study mentioned that the International Labour Organization has found more than 50 definitions in 75 different countries [18]. However, the European Commission stated that enterprises can be defined based on the number of employees, annual turnover, and financial criteria. The World Bank has used three criteria to define SMEs: number of employees, annual sales in U.S. dollars, and total assets in U.S. dollars.

The World Bank conducted a study across 132 countries in order to define SMEs. A third of the countries (64 out of 132) defined SMEs as businesses that have fewer than 250 employees, but every country has the freedom to define SMEs according to their needs [19].

Saudi Arabia established Monshaat in 2016. The objectives of Monshaat are to organize, support, develop, and sponsor the SME sector in the KSA in accordance with international best practices to raise the productivity of these enterprises and increase their contribution to the gross domestic product from 20% to 35% by 2030. Monshaat has identified three categories for SMEs: micro, small, and medium. SMEs are categorized according to two criteria: the number of full-time employees and the total volume of revenue [20]. Table 1 shows the categorization of SMEs according to Monshaat.

One of the sectors most affected by cyberattacks is small and medium-sized enterprises (SMEs). The reason behind the increasing attacks on SMEs is the weakness of their infrastructures compared to those of large enterprises [9]. SMEs regularly have difficulties in complying with new regulations and deploying security measures in their systems and hardware due to lack of resources, lack of experience, and lack of awareness [9]. A report indicated that 93% of SMEs have been affected by cybersecurity incidents that caused a financial loss [21]. Fifty percent of SMEs have faced a problem in operating their businesses because of cybersecurity issues. Thirty-one percent have seen their reputations damaged, causing a loss of customers. The report indicated that cyberattacks are increasingly targeting SMEs and that 50% of all cyberattacks in 2017 were against SMEs. Cybersecurity can heavily disrupt SMEs’ business, negatively impacting the financial bottom line, and it can be difficult to recover from such incidents. There are two reasons why an SME may be targeted: the SME does not have strong and robust security, or the SME does not invest sufficiently in security relative to large enterprises [21].

In 2017, the Office of the New South Wales Small Business Commissioner estimated the average cost of a cybersecurity event was $50,000 per incident and that 60% of SMEs would be affected by a cybersecurity incident [22]. Another study was conducted by [5] to identify the state of cybersecurity among SMEs in the United States and the United Kingdom. The study encompassed 1045 individuals from different companies and showed that the percentage of cyberattacks targeting SMEs had increased from 61% in 2017 to 67% in 2018. The average cost for companies to recover also increased from $1.03 million in 2017 to $1.43 million in 2018. The average cost of returning to normal operations after the cyberattack increased from $1.21 million in 2017 to $1.56 million in 2018 [5].

Even so, many SMEs do not understand how to protect themselves from cyberattacks, which further contributes to SMEs coming under attack [17,23] especially when the attackers take advantage of some security vulnerabilities such as DNS typo-squatting attacks as mentioned by [24].

## 3. Research Framework

The aim of this research is to determine the impact of various security practices on the damage caused by cybersecurity attacks, especially for small enterprises in Saudi Arabia. The selection of the dependent and independent variables was based on the cybersecurity literature [11,25]. Three aspects were selected to measure the damage caused by cybersecurity attacks, namely financial damage, loss of sensitive data, and restoration time. These aspects were selected to be the dependent variables in our model and they cover both direct and indirect impacts of cybersecurity attacks as described by [11]. Additionally, 12 security practices were selected as independent variables, which are:

1. Cybersecurity awareness: this variable refers to the level of awareness of cybersecurity threats that small enterprises may face.
2. Knowledge of cybersecurity damage: this variable refers to the level of knowledge about the damage that could be caused by cybersecurity attacks.
3. Cybersecurity governance: this variable relates to cybersecurity governance practices at the enterprise.
4. Application of cybersecurity policies: this variable refers to how cybersecurity policies are applied at the enterprise.
5. Use of protection systems: this variable relates to the use of any protection systems at the enterprise.
6. Following cybersecurity procedures: this variable measures how closely cybersecurity procedures are followed at the enterprise.
7. Specialized training in cybersecurity: this variable refers to any specialized training in cybersecurity that employees of the enterprise receive.
8. Contact with cybersecurity authorities: this variable describes whether the enterprise contacts the authorized cybersecurity organizations in the country after a cybersecurity event.
9. Inspection team: this variable refers to the availability of a special team to investigate cybersecurity threats at the enterprise.
10. Recovery plan: this variable refers to the presence or absence of a recovery plan for dealing with cybersecurity attacks at the enterprise.
11. Protection software pricing: this variable measures the expensiveness of the enterprise’s protection software.
12. Professionals’ salaries: this variable measures the cybersecurity professionals’ salaries.

**Figure 2:**
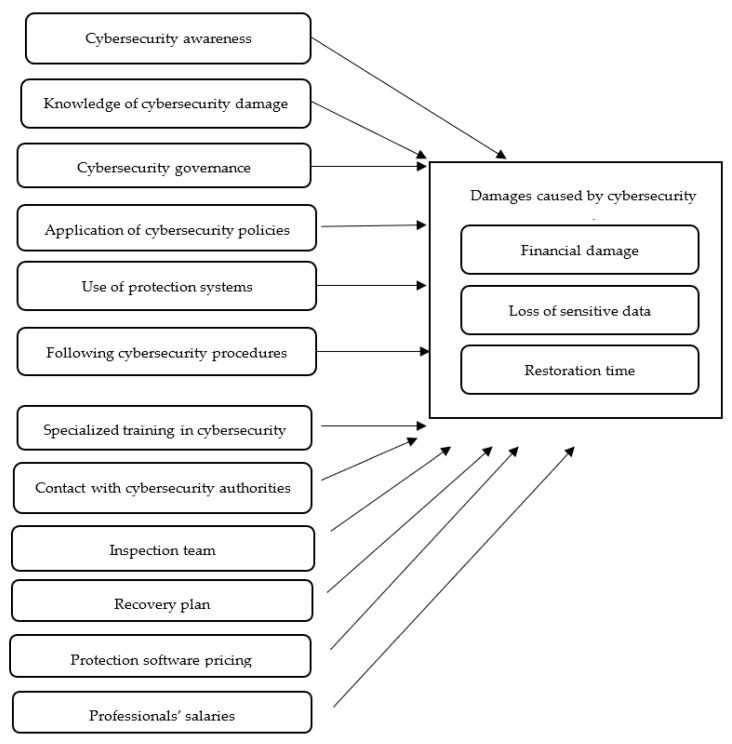
Research framework.

In order to understand the relationship between dependent and independent variables, a research framework is proposed as shown in Figure 2.

## 4. Research Method

This study aims to measure the effectiveness of security practices at small enterprises in Saudi Arabia in the event of a cybersecurity attack. Thus, a questionnaire was developed targeting a variety of stakeholders in small enterprises in Saudi Arabia.

A literature review was conducted to identify the items to include in the questionnaire that was then developed. Subsequently, the questionnaire was reviewed by a panel of experts in the computer science department of Shaqra University to check the validity of the content. The questionnaire was then translated into Arabic by the authors and reviewed by an expert to check the quality of the translation. The pilot study was conducted with a group of master’s degree students to identify any spelling or timing issues. The researchers obtained ethical approval for this research from the Research Ethics Committee at Shaqra University in Saudi Arabia.

Google Forms was implemented as an online survey tool. The subjects of this research consisted of various stakeholders who are involved in small enterprises in Saudi Arabia such as employees, customers, owners, supporters, or partners. The researchers sent the link via email to the participants and applied the snowball sampling technique to reach more participants. Although the snowball sampling technique has the possibility of the respondents sharing the same characteristics, the researchers try to select the initial participants carefully and with a diversity of roles to avoid such limitations [26].

The questionnaire consisted of 19 questions and was divided into four sections. The first section included information about the study and research team and provided the consent form. The second section collected demographic information about the participants such as gender, age, and their role in the small enterprise. The third section included the factors being examined to measure their impact on the damage caused by cyberattacks on small enterprises in Saudi Arabia. The fourth section encouraged participants to leave additional comments regarding the study.

## 5. Data Analysis

A total of 296 respondents, all of them involved in small enterprises in Saudi Arabia, responded to the survey during the period from 3 December 2020, through 18 March 2021. However, the responses of 14 participants were not included in the analysis as they were incomplete. Thus, 282 participants completed the online survey. The analysis was conducted using the statistical package for the social sciences in IBM SPSS version 27.

### 5.1. Sample Characteristics

While more than 70% of the participants are male, only 29.8% are female. Of the respondents, 62.1% are 35 years old or younger, and 37.9% are older than 35 years old. The survey includes participants with various roles at the small enterprises: 35.1% are employees, 22.3% are customers, 19.5% are owners, 17.8% are supporters, and 5.3% are partners. Table 2 represents the demographic characteristics of the respondents.

### 5.2. Experience of Cybersecurity Attacks

The results indicate that 14.2% of the participants reported financial damages to their small enterprise as a result of a cyberattack. Additionally, 20.5% of the participants reported that their enterprises lost sensitive data during a cyberattack. For the majority of participants (50.3%), the restoration time for their enterprises’ systems after a cybersecurity attack was days or less. However, for some participants (9.6%), the restoration took months. Table 3 presents the data on the respondents’ experiences with cybersecurity attacks.

### 5.3. Measurement Models

The participants were asked to answer questions that measured the dependent and independent variables as shown in the research framework. Appendix A Table A1 shows the respondents’ answers to the questions. The value of Cronbach’s Alpha for the independent variables was 0.692, which is considered an acceptable value [27].

Multiple regression analysis was conducted on the factors identified in the literature review in order to understand how much they affected the level of damage caused by cybersecurity attacks with a statistical significance level of 5% (i.e., a confidence level of 95%). The regression analysis indicated whether there was a correlation, positive or negative, between each factor and the level of damage caused [28]. The outcomes of the multiple regression analysis are as follows:

#### 5.3.1. Financial Damage

All independent variables were inserted into the multiple regression analysis test and the model showed a moderate level of prediction, the model quality measured by the multiple correlation coefficient “R” being 0.382, as seen in Table 4.

The calculated F-ratio seen in Table 4 of 3.519 indicates that the overall regression model is a good fit for the data as the dependent variables were statistically significant.

Table 5 shows the model coefficients of the independent variables and their relationships with the dependent variable of financial damage.

The model indicates that only the independent variables of having an inspection team and having a recovery plan had statistically significant effects on the financial damage resulting to the small enterprises. The negative sign indicates a negative relationship between the dependent and independent variables, meaning that small enterprises that have an inspection team and a recovery plan will likely suffer less financial damage.

#### 5.3.2. Loss of Sensitive Data

Independent variables were tested through multiple regression analysis to determine whether they have an impact on the loss of sensitive data in small enterprises. Table 6 shows that the model has a moderate level of quality as the multiple correlation coefficient calculated, “R”, was 0.312.

The F-ratio of 2.229 shown in Table 6 indicates that the overall regression model is a good fit for the data as the dependent variables were statistically significant.

Table 7 shows the model coefficients of the independent variables and their relationships with the dependent variable of loss of sensitive data.

The model shows that only the factors of cybersecurity awareness, knowledge of cybersecurity damage, and professionals’ salaries had a statistically significant effect on the loss of sensitive data at small enterprises. The positive relationship between the knowledge of cybersecurity damage and data loss indicates that the existence of this knowledge increases the likelihood of losing sensitive data at the small enterprise. The negative relationship between cybersecurity awareness and the loss of sensitive data in the small enterprise means that as the small enterprise’s employees become more aware of cybersecurity, the possibility of losing sensitive data is minimized. Finally, professionals’ salaries have a negative effect on the loss of data in small enterprises, which may indicate that as professionals’ salaries increase, the chance of losing sensitive data at the small enterprises decreases.

#### 5.3.3. Restoration Time

The independent variables were tested through multiple regression analysis to indicate whether they have an impact on the variable of restoration time in small enterprises. Table 8 shows that the model has a moderate level of quality as the calculated multiple correlation coefficient “R” was 0.369. The table also shows that the calculated F-ratio of 3.248 is statistically significant. This value indicates that the overall regression model is a good fit for the data as the dependent variables were statistically significant.

Table 9 shows the model coefficients of the independent variables and their relationships with the dependent variable.

The results shown in Table 9 indicate that contact with cybersecurity authorities and the presence of inspection teams had statistically significant effects on restoration time at small enterprises. Both independent variables can be seen to have a negative effect on the restoration time, which means that when a small enterprise has a sound policy in place for establishing emergency contact with authorities in the event of a cyberattack, the time it takes to restore data is minimized. Additionally, the existence of an inspection team may contribute to minimizing data restoration time as well.

## 6. Discussion

This study aims to identify what factors may impact the level of damage done by cybersecurity attacks on small enterprises in Saudi Arabia in terms of three different aspects, namely financial damage, the loss of sensitive data, and the length of time required to restore the system to its normal functioning. While 20.5% of respondents stated that their organizations had lost sensitive data during the attacks, only 14.2% of the participants reported that the cybersecurity attack caused financial damage to their enterprises. The results are similar to findings from the Australian Competition and Consumer Commission (ACCC) [23], which indicated that 26.4% of small-sized organizations in Australia faced financial harm due to cybersecurity events. The amount of time it took SMEs in this study to restore their systems to normal functioning varied between enterprises and reached days (22.3%) or months in some cases (9.6%). This is considered a long time when compared with other enterprises, as mentioned in [29].

This study also aims to discover the impact certain cybersecurity practices have on the three above-mentioned aspects.

The results indicate that only two factors—inspection team and recovery plan—have an impact on the financial damage caused by cybersecurity attacks on small enterprises. The multiple regression analysis shows that small enterprises that have an inspection team and a recovery plan are less likely to suffer major financial damage in the event of a cybersecurity attack. This result shows the importance of having a dedicated team in place to review the procedures related to information security measures, as required by many authorities in Saudi Arabia, such as the Capital Market Authority [30]. Having a recovery plan at the ready is also recommended by many international organizations such as the International Organization for Standardization and International Electrotechnical Commission (specifically, ISO/IEC 27031) [31] as well as the National Cybersecurity Authority (NCA) in Saudi Arabia [32].

Regarding the loss of sensitive data, three factors were identified as having an impact: cybersecurity awareness, knowledge of cybersecurity damage, and professionals’ salaries. It was surprising to find a positive relationship between the knowledge of cybersecurity damage and the loss of sensitive data among small enterprises. However, a possible explanation of this finding may be that when employees have the cybersecurity knowledge necessary to identify data security breaches, more such breaches will be reported [33]. Many studies have indicated the effectiveness of cybersecurity awareness in reducing the impact of cybersecurity attacks [9]. Professionals’ salaries were also found to have an impact on the loss of sensitive data from cybersecurity attacks; specifically, enterprises that provided higher salaries were less likely to lose sensitive data. The positive relationship between economic incentives and improved levels of cybersecurity was also identified in [34].

Only two factors, contact with cybersecurity authorities and having an inspection team, were found to have statistically significant effects on restoration time. Contacting the national cybersecurity authorities is compulsory in many cybersecurity frameworks, such as [35], especially in the event of mid-level or highly classified security breaches. Doing so could reduce the impacts of cybersecurity incidents and ensure that organizations are following the security protocols provided by the authorities. Having a security operations team that can inspect cybersecurity activity has also been recommended by many authorities [30,32,35]. Based on this study’s findings, the establishment of such a team can prove highly beneficial for small enterprises in the event of a cybersecurity attack.

## 7. Limitation

There are some limitations to the current study. The first limitation is that the sample is limited to small enterprises in Saudi Arabia. Studying the same enterprises in different countries can provide interesting information that can compare with the findings of our study. Another limitation is regarding the subjective nature of some questions in the survey. However, the researchers tried to reduce the impact of this issue by increasing the breadth of the statistical sample [36].

## 8. Research Implication

The current research provides many implications to the research community, managers, and cybersecurity practitioners. Firstly, the findings showed the importance of certain cybersecurity practices such as the availability of inspection teams and recovery plans at small enterprises. Thus, it is important for small enterprises to have effective inspection teams that can predict any cybersecurity vulnerabilities in their IT systems. Although this could not be available for all small enterprises due to the lack of resources, this can be achieved through cybersecurity services provided by IT vendors. The results also indicated that having a well-prepared recovery plan can be effective and reduce the required restoration time after cybersecurity attacks. Small enterprises should define their disaster recovery plan precisely to avoid long restoration time that can affect their business negatively [37]. Additionally, it is important for small enterprises to keep continuous training about new threats of cybersecurity as our study indicated that the knowledge of cybersecurity damage can help in discovering any loss of sensitive data. Our study also confirmed the vital role of cybersecurity authorities that can improve the practices of cybersecurity at small enterprises either by educating them or by forcing them to follow certain practices to reduce the impact of cybersecurity attacks.

## 9. Conclusions

As the importance of information and communication technologies has increased, the need to protect these technologies has increased likewise. Thus, cybersecurity is becoming a vital consideration for any organization. However, small enterprises still face difficulties in providing the required cybersecurity protection for various reasons such as the high cost of cybersecurity solutions. Our paper discusses the relationship between various cybersecurity practices and the damage caused by cybersecurity attacks, which is an emerging research topic. Twelve cybersecurity practices and three possible impacts of cybersecurity attacks were discussed and tested using multiple regression analysis. The results showed that having an inspection team and a recovery plan may limit the financial damage caused by cybersecurity attacks on small enterprises. The results also indicated that cybersecurity awareness, knowledge of cybersecurity damage, and professionals’ salaries were related to the loss of sensitive data. Furthermore, the results showed that contact with cybersecurity authorities and having an inspection team have statistically significant effects on restoration time. The implication of this study is that small enterprises should focus more on certain cybersecurity practices that can decrease the impacts of cybersecurity attacks. Future studies are suggested to overcome the limitation of this research. For instance, it would provide valuable insight to increase the sample population to include large organizations, apply a similar research framework to them, and compare the results with those of the current study.

## Figures and Tables

**Table 1:** Categorization of SMEs as defined by Monshaat in Saudi Arabia.

Enterprises	Number of Full-Time Employees	Volume of Revenue
Micro	1–5	SAR 0–3 Million
Small	6–49	SAR 3–40 Million
Medium	50–249	SAR 40–200 Million

**Table 2:** Demographic characteristics of participants.

Characteristic	Total Respondents	
	Frequency	Percent
Gender		
Male	198	70.2%
Female	84	29.8%
Total	282	100%
Age (years)		
18–25	67	23.8%
26–35	108	38.3%
36–45	71	25.2%
46 or older	36	12.7%
Total	282	100%
Role		
Employee	99	35.1%
Customer	63	22.3%
Owner	55	19.5%
Supporter	50	17.8%
Partner	15	5.3%
Total	282	100%

**Table 3:** Cybersecurity attacks.

Has a Cyberattack Ever Caused Financial Damage to Your Small Enterprise?		
Yes	40	14.2%
No	242	85.8%
Total	282	100%
Did the small enterprise lose sensitive data during a cyberattack?		
Yes	58	20.5%
No	102	36.2%
I do not know	122	43.3%
Total	282	100%
How long did it take to restore the systems to their normal state?		
Hours	79	28.0%
Days	63	22.3%
Months	27	9.6%
I do not know	113	40.1%
Total	282	100%

**Table 4:** Model summary (financial damage).

Model	R	R Squared	Adjusted R Squared	Std. Error of the Estimate	F	Sig.
1	0.382 ^a^	0.146	0.104	0.33077	3.519	0.000

**Table 5:** Model coefficients (financial damage).

	Std. Error	Standardized Coefficients Beta	T	Sig.
Constant	0.077		11.845	0.000
Cybersecurity awareness	0.050	−0.025	−0.367	0.714
Knowledge of cybersecurity damage	0.055	−0.121	−1.725	0.086
Cybersecurity governance	0.059	0.015	0.203	0.840
Application of cybersecurity policies	0.041	−0.028	−0.474	0.636
Use of protection systems	0.032	0.072	1.051	0.294
Following cybersecurity procedures	0.015	−0.026	−0.366	0.715
Specialized training in cybersecurity	0.061	0.043	0.702	0.483
Contact with cybersecurity authorities	0.051	−0.042	−0.615	0.539
Inspection team	0.054	−0.159	−2.057	0.041 *
Recovery plan	0.054	−0.212	−2.717	0.007 *
Protection software pricing	0.027	0.091	1.513	0.131
Professionals’ salaries	0.029	0.051	0.813	0.417

* Significant at the 0.05 level.

**Table 6:** Model summary (loss of sensitive data).

Model	R	R Squared	Adjusted R Squared	Std. Error of the Estimate	F	Sig.
1	0.312	0.098	0.054	0.312	2.229	0.009

**Table 7:** Model coefficients (loss of sensitive data).

	Std. Error	Standardized Coefficients Beta	T	Sig.
Constant	0.201		60.326	0.000
Cybersecurity awareness	0.131	−0.165	−2.389	0.018 *
Knowledge of cybersecurity damage	0.144	0.157	2.182	0.030 *
Cybersecurity governance	0.154	0.020	0.270	0.787
Application of cybersecurity policies	0.107	−0.024	−0.397	0.692
Use of protection systems	0.083	−0.064	−0.913	0.362
Following cybersecurity procedures	0.039	0.014	0.188	0.851
Specialized training in cybersecurity	0.161	0.083	1.330	0.185
Contact with cybersecurity authorities	0.134	0.084	1.198	0.232
Inspection team	0.141	−0.061	−0.770	0.442
Recovery plan	0.143	−0.092	−1.145	0.253
Protection software pricing	0.070	−0.051	−0.821	0.412
Professionals’ salaries	0.075	−0.183	−2.863	0.005 *

* Significant at the 0.05 level.

**Table 8:** Model summary (restoration time).

Model	R	R Squared	Adjusted R Squared	Std. Error of the Estimate	F	Sig.
1	0.312	0.098	0.054	0.312	2.229	0.009

**Table 9:** Model coefficients (restoration time).

	Std. Error	Standardized Coefficients Beta	T	Sig.
Constant	0.202		14.312	0.000
Cybersecurity awareness	0.132	−0.116	−10.711	0.088
Knowledge of cybersecurity damage	0.144	−0.081	−10.154	0.249
Cybersecurity governance	0.155	0.004	0.059	0.953
Application of cybersecurity policies	0.107	0.016	0.268	0.789
Use of protection systems	0.083	−0.039	−0.575	0.566
Following cybersecurity procedures	0.039	−0.030	−0.422	0.673
Specialized training in cybersecurity	0.161	0.010	0.164	0.870
Contact with cybersecurity authorities	0.134	−0.155	−2.269	0.024 *
Inspection team	0.142	−0.168	−2.159	0.032 *
Recovery plan	0.143	0.002	0.020	0.984
Protection software pricing	0.070	−0.012	−0.196	0.845
Professionals’ salaries	0.076	−0.025	−0.398	0.691

* Significant at the 0.05 level.

## Data Availability

The data presented in this study are available on request from the corresponding author. The data are not publicly available due to Shaqra University code of ethic.

## Appendix A

**Table A1:** Cybersecurity Practices.

Characteristic	Total Respondents	
	Frequency	Percent
Do you have cybersecurity awareness?		
Yes	190	67.4%
No	92	32.6%
Total	282	100%
Do you have knowledge of the extent of the damage caused by cyberattacks on small enterprises?		
Yes	206	73%
No	76	27%
Total	282	100%
Is there cybersecurity governance within the small enterprise?		
Yes	213	75.5%
No	69	24.5%
Total	282	100%
Do you apply cybersecurity policies within the small enterprise?		
Yes	142	50.4%
No	140	49.6%
Total	282	100%
Do you use any protection system in your small enterprise?		
Yes	234	83%
No	48	17%
Total	282	100%
Do your workers follow cybersecurity procedures with all devices in the small enterprise?		
Yes	171	60.6%
No	60	21.3%
When problems occur	51	18.1%
Total	282	100%
Does the small enterprise instill cybersecurity awareness in employees?		
Yes	203	72%
No	79	28%
Total	282	100%
Do the small enterprise’s employees receive specialized cybersecurity courses?		
Yes	243	86.2%
No	39	13.8%
Total	282	100%
When the small enterprise is exposed to a cyberattack, is the cybersecurity authority contacted?		
Yes	194	68.8%
No	88	31.2%
Total	282	100%
Is there an inspection team to track cyber threats in the small enterprise?		
Yes	134	47.5%
No	148	52.5%
Total	282	100%
Does the small enterprise have a plan to recover from cyberattacks?		
Yes	150	53.2%
No	132	46.8%
Total	282	100%
Is it expensive to purchase and maintain protection software for the small enterprise?		
Yes	108	38.3%
No	78	27.7%
Sort of	96	34.0%
Total	282	100%
Are cybersecurity professionals’ salaries expensive at the small enterprise?		
Yes	114	40.4%
No	108	38.3%
Sort of	60	21.3%
Total	282	100%
